# Risk assessment of driver performance in the oil and gas transportation industry: Analyzing the relationship between driver vigilance, attention, reaction time, and safe driving practices

**DOI:** 10.1016/j.heliyon.2024.e27668

**Published:** 2024-03-11

**Authors:** Al-Baraa Abdulrahman Al-Mekhlafi, Ahmad Shahrul Nizam Isha, Nicholas Chileshe, Ahmed Farouk Kineber, Muhammad Ajmal, Abdullah O. Baarimah, Al-Hussein M.H. Al-Aidrous

**Affiliations:** a Faculty of Leadership and Management, Universiti Sains Islam Malaysia (USIM), Nilai, Malaysia; b Department of Management & Humanities, Universiti Teknologi PETRONAS, Seri Iskandar 32610, Perak, Malaysia; c UniSA STEM, Scarce Resources and Circular Economy (ScaRCE), University of South Australia, Adelaide 5001, Australia; d Department of Civil Engineering, College of Engineering in Al-Kharj, Prince Sattam Bin Abdulaziz University, Al-Kharj, 11942, Saudi Arabia; e Department of Civil and Environmental Engineering, College of Engineering, A'Sharqiyah University, 400 Ibra, Oman

**Keywords:** Risk assessment, Oil and gas transportation, Road safety, Driving performance, Driver attention, Vigilance, Reaction time

## Abstract

The increasing use of road traffic for land transportation has resulted in numerous road accidents and casualties, including those involving oil and gas tanker vehicles. Despite this, little empirical research has been conducted on the factors influencing tanker drivers' performance. This study aims to address this knowledge gap, particularly in the energy transportation industry, by examining the driving performance factors that affect tanker drivers and incorporating risk assessment measures. The model variables were identified from the literature and used to develop a survey questionnaire for the study. A total of 307 surveys were collected from Malaysian oil and gas tanker drivers, and the driving performance factors were contextually adjusted using the Exploratory Factor Analysis (EFA) approach. The driving performance model was developed using partial least squares structural equation modeling (PLS-SEM). The EFA results categorized driving performance into two constructs: 1) drivers' reaction time with β = 0.320 and 2) attention and vigilance with β value = 0.749. The proposed model provided full insight into how drivers’ reaction time, attention, and vigilance impact drivers' performance in this sector, which can help identify potential risks and prevent accidents. The findings are significant in understanding the factors that affect oil and gas drivers' performance and can aid in enhancing oil and gas transportation management by including effective risk assessment measures to prevent fatal crashes.

## 1 Introduction

Road crashes represent approximately 30% of the world’s fatal accidents [1]. The World Health Organization WHO [2] reports that over 1.35 million people die yearly from road crashes, and millions sustain severe injuries. 70 % of these crashes occur in third-world nations [3]. Although the data from 215 countries indicated 792 to 905 thousand worldwide road fatalities in 2014, the estimation models show a global expected decline of 12% in road fatalities by 2025 [4]. Heavy vehicle crashes are considered among the most dangerous road accidents because of the significant consequences of fatal injuries and economic losses. However, there are contextual variances in the severity of vehicle truck crashes [5]. Hosseinzadeh, Moeinaddini [6] examined factors impacting the gravity of truck-involved accidents and suggested that decreeing and imposing full rules concerning heavy vehicle drivers' working timetables and continuous observation by authorities can suggestively lessen large truck-involved accidents. Furthermore, the significant exploratory factors that reduce truck crashes provide new insights into the recognized factors that help minimize truck accidents [7].

As in many parts of the world, transportation crashes are a significant problem in Malaysia. The Royal Malaysia Police [8] reported that more than 6000 fatalities resulted from road traffic crashes in 2019. Analysis of road characteristics and environmental factors connected to fatal crashes in Malaysia showed that fatalities depend on the size of the vehicle involved [9]. Evaluation of the attributes of heavy goods vehicle crashes in Malaysia by Hamidun, R Hoong [10] indicates that crashes involving heavy vehicles on the highway are more likely when equated to other road types. The heavy vehicle accident along the Malaysian expressway led to over 80% of other vehicle fatalities [10]. These heavy vehicles' large dimensions and mass contribute to their gravity in fatalities of road accidents and vehicles involved [11]. Out of 7026 heavy vehicle accidents analyzed in Malaysia, 3787 cases were fatal, and 3239 were nonfatal, with severe and minor damage to drivers and passengers [10]. It indicates a significant impact of heavy vehicle accidents on the safety of other road users. Therefore, accidents involving heavy vehicles are crucial issues that require a deep understanding of the nature of these vehicles, their drivers, and the threat they pose to the life and safety of other road users [12].

The literature showed that truck crashes are a significant part of the Malaysian crash picture. Therefore, it is essential to understand the etiology of these crashes. For instance, Shankar, Mannering [13] identified several factors contributing to traffic crashes, including driver characteristics, environmental conditions, type of accident, vehicle attributes, and motorway design. In addition, many studies indicated that individual factors such as health, fatigue, age, and stress play a significant role in road accidents [[14], [15], [16], [17]]. Likewise, analysis of temporal forms of driving exhaustion and driving performance revealed the existence of recovery and lagging effects of temporal patterns. In addition, demographic variables, including net income and driver age, significantly correlate with the measured driving performance and fatigue [18]. Thus, it may be argued that human factors are the most significant impact causing the high rate of traffic road accidents [19,20]. Driving performance errors, such as diminished reaction time to surprise events, are an often-cited crash factor. These drivers’ errors can be attributed to various causes, including reduced vigilance (e.g., caused by fatigue) and driver distraction. Multilevel analysis of the role of human factors in disparities in accident outcomes indicated that human-centered reasons affect severe damage accident rates [21].

Consequently, this research aims to analyze heavy vehicle drivers' low-performance causes regarding human factors in the Malaysian energy sector. Thus, exploratory factor analysis (EFA) proposes a quantitative survey approach. Likewise, partial least square structural equation modeling PLS-SEM was employed to test these factors in the Malaysian oil and gas industry. Furthermore, this study adopts the Global-Local Context, which further illustrates the study's international significance based on the local investigation. The approach signifies the problems examined and generalizes their results. Thus, the study was conducted in the Malaysian oil and gas transportation sector. The results from this research can help obtain a picture of the driver's performance and road safety in developing countries, especially in the oil and gas transportation sector.

Overall, it can be concluded that several studies have investigated the essential factors that influence driving performance. However, there is a need to explore the driving performance indicators, which have not been thoroughly investigated, particularly among oil and gas tanker drivers. This study reports a novel effort to understand the driving performance factors among tanker drivers and gain more insights into the oil and gas industry. In addition, this study contributes to the oil and gas transportation industry with a better comprehension of driving performance measures. It also gives a strong vision for managing transportation companies that want to achieve their obligations with zero accidents. Moreover, the present analysis can assist the parties involved, such as managers, supervisors, and drivers, as well as concentrate on drivers' vigilance and attention throughout oil and gas transportation responsibilities.

### 1.1 Motivation for this study

This study aims to investigate the causes of low driving performance among heavy vehicle drivers in the Malaysian energy sector, specifically in the oil and gas transportation industry. The study is driven by the significant impact of heavy vehicle accidents on road safety, as well as the high rate of traffic accidents in Malaysia, which result in many fatalities and severe injuries. The literature review identifies several factors contributing to road crashes, including driver characteristics, environmental conditions, type of accident, vehicle attributes, and motorway design, with human factors being the most significant impact.

The contribution of this study lies in its attempt to understand the driving performance factors among tanker drivers and gain more insights into the oil and gas industry. It also provides a better comprehension of driving performance measures for the oil and gas transportation industry, which can assist in achieving zero accidents. Moreover, the study can help transportation companies' management and drivers to concentrate on drivers' vigilance and attention throughout oil and gas transportation responsibilities. The study's results can also help understand driver performance and road safety in developing countries, particularly in the oil and gas transportation sector. Therefore, the study has international significance based on local investigation, as it illustrates the problems examined and generalizes their results.

## 2 Research background

This research comprehensively reviewed the relevant literature using two primary databases, Google Scholar and Scopus. These databases were chosen for their extensive coverage of scholarly publications across various disciplines. To identify pertinent articles, we employed the following search terms: “driving performance,” “driver attention,” “vigilance,” and “reaction time.” These keywords were carefully selected to encompass the key aspects of our research topic, ensuring that we capture a wide range of relevant studies. Furthermore, we established specific criteria for the selection of articles. We focused on peer-reviewed publications, including journal articles and conference papers, to ensure the reliability and credibility of the sources. Additionally, we considered articles published within the past five years to ensure the relevance and timeliness of the research.

Our search strategy involved reviewing abstracts and full texts, where necessary, to determine the inclusion of articles in our scoping review. During this selection process, we also assessed each article's methodology, findings, and relevance to our research objectives.

Performance is completing a particular task as assessed against recognized precision, completeness, cost parameters, and speed. Driving performance is the effectiveness of completing driving duties [22]. In the oil and gas sector, safety is one of the essential things that needs to get high attention [23] due to road crashes accelerating daily. Oil and gas tanker drivers performance is deemed to be fulfilled an obligation and duties [[24], [25], [26]]. Driving performance is attention [27]; Drivers frequently split their attention between driving tasks like lane position management and speed and other activities like dealing with dashboard electronics. The increasing amount of “in-car technology” lead to distractions (such as entertainment systems, cellphones, and navigation system), diverting the attention of drivers is especially concerning. As a result, these distractions have been identified as one of the critical reasons for poor performance [[28], [29], [30]].

Several studies have explored risk factors linked with driving performance [31,32]. According to Kee, Tamrin [33], driving performance deteriorated dramatically during the long driving period when considering some driving conditions and environmental factors. Divided attention negatively impacted driver performance, particularly under alcohol [27]. Larue, Rakotonirainy [34] demonstrated that decreased vigilance impairs driving performance because of road design and roadside variability; this is called a monotonous road environment, which leads to boredom with drivers during their duties. These impairments were also linked to the observed driver, engine, and environmental measurements.

Similarly, Adrian, Postal [35] conducted cognitive tests involving forty-two participants aged 60 and above. Results revealed a significant relationship between personality traits and executive functions, with low driving performance. Mets, Ketzer [36] indicate that energy drink increases driving performance significantly and decreases driver sleepiness when on a prolonged highway. Brooks, Crisler [37] indicated that driving in fog with high speed significantly degraded performance. Safety advantages can be achieved by persuading drivers to slow down rather than speed driving in fog conditions.

Additionally, Alosco, Spitznagel [38] conducted a study among university students in Ohio, United States, indicating that texting and eating during driving are related to low driving performance. However, Ünal, Steg [39] attempted to examine how loud music influences performance and how mental effort can mediate this impact. Based on the results, loud music increases mental effort, supporting the general opinion that music can distract. Likewise, Oviedo-Trespalacios, Haque [40] meta-analysis showed that mobiles significantly impact low driving performance. Perlman, Samost [41] investigated the impact of the smartwatch in initiating telephone calls on driver performance. Results suggest that visual manual calling has a more significant influence on driving performance than the voice calling methods for the interfaces tested. Such results indicate that the smartwatch is one of the reasons that distracts drivers, leading to low attention of drivers. Otherwise, vehicular networking is quite related to driver behavior and performance [[42], [43], [44]].

Thus, this study explores the driving performance factors among tanker drivers to gain insights from the oil and gas transportation industries. Most heavy vehicle drivers are subjected to different traffic regulations, and their companies observe safety performance while discharging responsibilities. Nevertheless, truck drivers are in total control of their actions. Therefore, speeding or reckless driving are consequences of their manner, which lead to such behavioral actions. Therefore, exploring the driving performance factors among tanker drivers is essential for the improved safety of truck drivers and other road users.

The oil and gas transportation sector greatly impacts the economy, society, and the environment, linking to sustainability [24]. A model that increases the driver's awareness of road safety needs to be developed and provides a clear vision toward safer transportation [14]. Drivers should remain attentive during their driving tasks to improve road safety. Previous studies on driving performance have focused mainly on distraction factors that impact drivers' performance [31,45,46]. Choudhary, Pawar [47] showed a lack of reflection of incidental effects underestimating accident risk. Likewise, operating music players and texting has led to more crash risks. Although driving distraction happens among long-haul truck drivers, it is also apparent that visually demanding jobs pose the most danger when compared to other sorts of work [48]. Driver mental distraction discovery using driving performance modeling showed that although the understanding of these models is low, it is satisfactory since the driver might control the vehicle adequately [49]. Examining the impact of human variables on road safety, emphasizing risk perception and risk-taking among new drivers, revealed a positive relationship between driving habits and risk perception. It is suggested that the more the driving years, the greater the risk perception. Likewise, a new driver's behavior hangs on many factors, including emotional state and social customs like peer gravity. This influence can intensify their disposition to take more risks [50]. In this study, driving performance has been evaluated from two viewpoints, driver reaction time and driver vigilance and attention, based on the results of the EFA analysis.

In summary, the above literature review examines driving performance in the oil and gas transportation sector, where safety is a critical concern. The review highlights the impact of distractions on driver performance, such as in-car technology, alcohol, fatigue, and personality traits. It also discusses the effect of environmental factors such as monotonous road design and roadside variability on driving performance. Other factors, such as loud music, energy drinks, and smartwatches, are also identified as distractions that affect driving performance negatively. The review suggests that exploring driving performance factors among tanker drivers is essential to improve road safety for truck drivers and other road users. It calls for developing a model that increases driver awareness of road safety situations and provides a clear vision towards safer transportation. The review also emphasizes the importance of risk perception and risk-taking among new drivers and the impact of emotional states and social customs on their driving behavior.

## 3 Methods

**Fig. 1:**
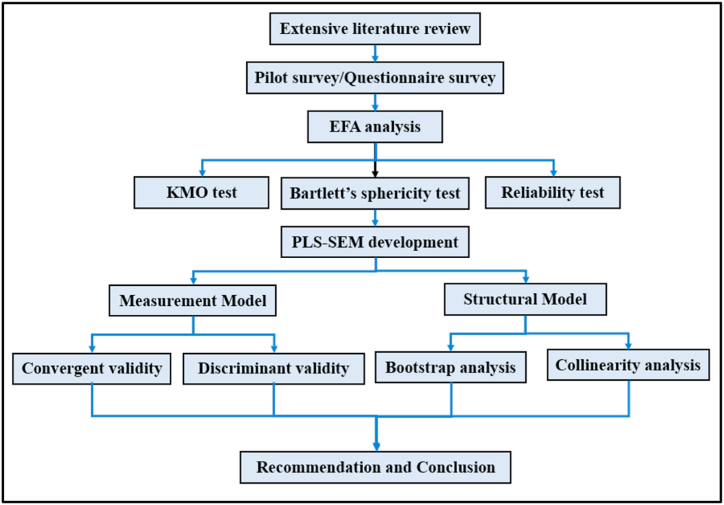
Research design.

Fig. 1 summarizes the phases of this study. It starts with an extensive literature review that identifies the causes of road crashes along highways. The questionnaire was developed and subjected to many validation stages based on the literature review. First, the content and face validity were assessed by an academic expert. Second, a pilot study was conducted to test reliability and internal consistency. Finally, the data were collected using the validated main questionnaire.

Subsequently, a pilot study, EFA analysis, construct validity analysis, and analytical analysis model (PLS-SEM) were undertaken. Finally, the measurement model and structural model are formulated. The former covers convergent and discriminant validity and comprises bootstrap and collinearity analyses. The participants were drivers who worked in the oil and gas transportation industry in different regions of Malaysia. Three hundred and fifty-seven (357) tanker drivers participated in this survey. This method involved dividing the driver population into relevant subgroups based on company location and randomly selecting representatives from each subgroup. This approach ensured our sample represented the diversity within the tanker driver population while maintaining fairness and objectivity. The data collection was done personally, with a useable response rate of 85.9%. Three hundred and seven (307) surveys were employed for Exploratory Factor Analysis and Partial least square-structural equation modeling analyses [51].

In sociodemographic terms, the survey exhibited a pronounced gender imbalance, with 99.7% males and 0.3% females, mirroring the male-dominated nature of the Malaysian oil and gas transportation sector, particularly in heavy vehicle operations. The age distribution skewed towards middle-aged and young individuals, with 14.7% aged 20–29, 48.2% aged 30–39, 26.4% aged 40–49, and 10.1% old 50–59, while only 0.7% aged 60 or above. Marital status revealed 84.4% were married, 12.7% single, and 2.9% separated respondents. The educational background showed 83.7% with secondary education, 12.7% with college diplomas, 2.6% with primary education, and 1% with graduate/postgraduate education, reflecting a strong presence of secondary education, in line with the sector's employment criteria.

### 3.1 Survey questionnaire

The questionnaire was utilized to collect data personally from tanker drivers to examine and validate driving performance factors. These factors were categorized based on the existing literature adopting quantitative research and examined using a survey. Driving performance factors were identified from the literature [52,53]. These items reflect attention, reaction time, and driver's vigilance. Attention sample items are “Operating entertainment systems do not distract me from driving (e.g., playing radio)" and “Operating navigation systems do not distract me from driving”. The sample items of reaction time are " My reactions are faster than they used to be (e.g., braking in an emergency)" and " I sometimes cannot judge my speed”. Lastly, the sample items' driver vigilance is " I sometimes cannot hear the horns of other vehicles/sirens from emergency vehicles”, and " Sometimes my speedometer is hard to read during the daytime".

This survey helped to evaluate: (i) the behaviors of drivers on the performance view and (ii) the connection between different variables, in particular, the linkage between causes and effects [54]. The testing method (questionnaire) was first suggested by Fellows and Liu [55]. It was used to test the questionnaire's understandability, ease of answer, and clarity, create the questionnaire, and establish the time necessary for answering it. Concerning the survey, driving performance factors are rated using a Likert scale (5-point) as often employed in literature [56].

Regarding study ethics, the Department of Management & Humanities at Universiti Teknologi PETRONAS provided ethical approval for this study under number YUTP-015LC0-043. As instructed, an introduction was added to inform participants about the study's goal and invite them to participate in this survey as volunteers. Besides, we obtained informed consent from all participants in the study and guaranteed them secrecy and anonymity.

### 3.2 Analysis of construct validity

For variables analysis, the Confirmatory Factor Analysis (CFA) and Exploratory Factor Analysis (EFA) procedures are commonly utilized [57]. In this study, EFA tests data about relations and variables and then reduces many variables into a few underlying structures. Meanwhile, CFA has been employed to assess the structure relationships underlying various variables in such theories or hypotheses [57]. The study used multivariate analytic approaches such as EFA to investigate the basic constructs of driving performance factors. It was employed to analyze the validity of constructs by evaluating the appropriateness, un-dimensionality, validity, and reliability of each measuring aspect of the various constructs (i.e., measurement models).

The required samples for EFA analysis should be between 150 and 300 for adequate factor analysis [58]. In the current study, eleven (11) variables and a sample size 307 are deemed sufficient for factor analysis [58].

### 3.3 Analytical analysis model (PLS-SEM)

To model the factors of driving performance, the Smart PLS version 3.2.7 has been used to apply the structural equation modeling PLS-ESM [59]. More attention has been paid in many fields to structural equation modeling, Partial least squares (PLS-SEM), notably in social sciences research [60]. PLS-SEM may be performed by analyzing the measurement model and the structural model. The measuring model exposes the items' present connections and their underlying latent variables. The structural model has been performed to test the path coefficient (β) and represent the inner relations between the eleven items [61]. SEM has previously been used in many areas, such as road safety [62] and the construction industry [[63], [64], [65], [66]]. The findings are provided in the section that follows.

## 4 Results

This study reports our novel effort to understand the driving performance factors among tanker drivers to gain more insights into the oil and gas industry.

### 4.1 EFA for driving performance factors

The factor ability structure of eleven (11) items linked to driving performance factors was determined using exploratory factor analysis (EFA). The Kaiser-Meyer-Olkin factor for homogeneity is widely utilized to ensure that partial correlations between study variables are as low as possible [67]. The KMO index runs from 0 to 1, with a minimum value of 0.6 for practical factor analysis [58]. Furthermore, Bartlett's sphericity assessment shows that the matrix in which p < 0.05 is significant is the matrix for the association [68,69]. In this study, the KMO sample adequacy measure is 0.879, above 0.6, which has been recommended, and Bartlett's sphericity test has shown to be significant = 5096.339, p < 0.05).

The diagonal matrix of the anti-image correlation is all greater than 0.5, implying that the inclusion of each variable in the component analysis is justified. For each factor, the initial commonalities are the variance estimates that all variables are considered. Values (0.3) are advised for variables that do not fit the factor solution. All starting groups in this study are over the threshold. The total loading factors are more than 0.5, which is deemed significant. The EFA analysis was utilized for all eleven (11) elements of driving performance, as indicated in Table 1. The findings revealed two components with eigenvalues greater than one. The overall variance indicated by the three components is 80.047 percent, whereas the total variance stated by the eight factors is 46.60 percent.

**Table 1:** Factor loading of driving performance factors.

Driving performance factors	Component	
	1	2
DP-1	0.706	–
DP-2	0.692	–
DP-3	–	0.919
DP-4	–	0.899
DP-5	0.681	–
DP-6	–	0.921
DP-7	0.732	–
DP-8	0.826	–
DP-9	0.846	–
DP-10	0.843	–
DP-11	0.844	–
Eigenvalues	7.6	1.2
% Of Variance	46.60	80.047

### 4.2 Measurement model

The SEM's measurement model for (driving performance factors) has assessed reliability, convergent validity, and discriminative validity. The structural model will be evaluated when the measurement model's reliability and validity have been proven [70]. According to Table 2, all constructions passed the AVE test. The optimal level of the AVE should be more than 0.5 [71]. Using the PLS algorithm, the AVE estimates for all constructs in this study are more than 0.50. Garver and Mentzer [72] state that alpha Cronbach values greater than 0.6 are appropriate for newly constructed measures. In contrast, the usual number is 0.7. As a result, values greater than 0.75 are deemed highly accurate. Consequently, the results are acceptable because the alpha Cronbach values are more than 0.6.

**Table 2:** Convergent validity findings.

Constructs	Item	Loading	Cronbach's Alpha	Composite Reliability	AVE
Reaction time	DP-3	0.987	0.979	0.986	0.960
	DP-4	0.964			
	DP-6	0.989			
Attention and vigilance	DP-2	0.845	0.984	0.956	0.733
	DP-5	0.835			
	DP-7	0.872			
	DP-8	0.835			
	DP-9	0.847			
	DP-10	0.871			
	DP-11	0.885			
	DP-1	0.857			

**Fig. 2:**
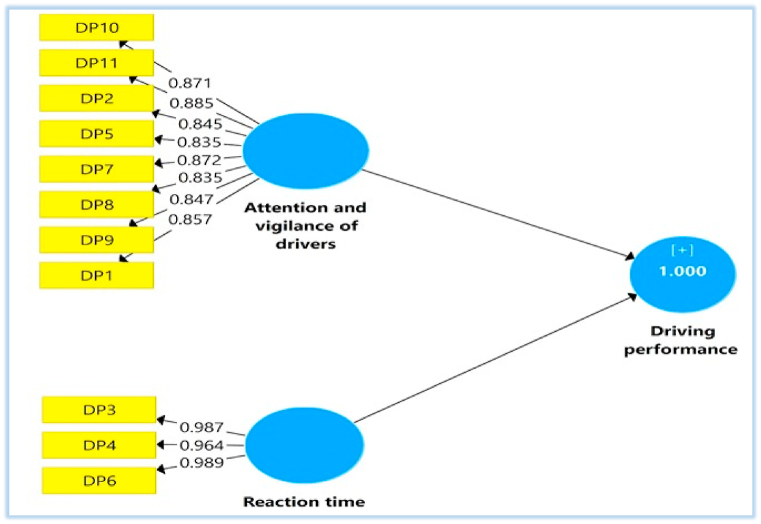
Measurement model.

The outer loadings for all measurement models fulfill the criteria of >0.70 and are thus acceptable [61]. Table 2 and Fig. 2 present the outer loading of the measurement models for all items. The total loadings are above 0.7 and thus acceptable. It means that each construct (group) measuring element is correctly measured, and no additional construct is assessed inside the study model. High outer loading on a structure implies that the respective elements of each structure have a close link. The general rule is that items with very low external loads (less than 0.4) often have to be taken off the scale [61]. These findings have shown that the measurement model is valid convergently and internally consistent.

In the cross-loading criteria, the discriminant validity was assessed. This technique seeks to establish that loading indications should exceed all other constructions per line in a latent construct. The value of indicators on the primary constructs must thus be higher than the charging on smaller constructs [70]. Table 3 shows that all latent (indicator) variables are loaded more frequently than other constructs. The results for each construct also demonstrate a significant degree of one-dimensionality.

**Table 3:** Cross loading results.

Items	Attention and vigilance of drivers	Reaction time
DP-10	**0.871**	0.589
DP-11	**0.885**	0.589
DP-2	**0.845**	0.625
DP-5	**0.835**	0.623
DP-7	**0.872**	0.649
DP-8	**0.835**	0.532
DP-9	**0.847**	0.533
DP-1	**0.857**	0.659
DP-3	0.691	**0.987**
DP-4	0.675	**0.964**
DP-6	0.697	**0.989**

### 4.3 Structural model

**Fig. 3:**
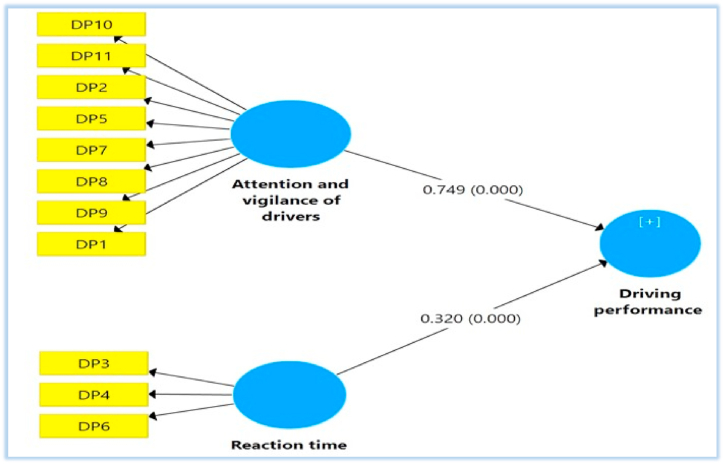
Structural model.

When determining the driving performance factors as a formative construct, the formative objects' collinearity is further studied by assessing the value of a variable inflation factor (VIF). Because subdomains contribute separately to higher-order structures, all VIF values are significantly lower than 3.5. In addition, the importance of the Path coefficients is predicted with a bootstrapping tool [73]. This study's paths are statistically significant (*P*-Value = 0.000), as illustrated by Fig. 3 and Table 4.

**Table 4:** Path analysis.

Paths	B	SE	P Values	VIF
Attention and vigilance - > Driving performance	0.749	0.01	0.000	1.97
Reaction time - > Driving performance	0.320	0.009	0.000	1.96

## 5 Discussion

Driver reaction time is the period from when the danger occurs to when the driver takes a specific action on the vehicle control to avoid an accident [74]. This study found that reaction time affects driving performance (β = 0.320, p < 0.000). The current finding agrees with Törnros [75] and Yoshida, Bolia [76]; similarly, another study found that reaction time was negatively impacted, which could have implications for driving performance [77]. The effect of driving speed on response time during highway driving revealed that the reaction time was slower at 70 km/h than at 110 km/h. However, there is no substantial difference in the driver's mood, basic response time, and concentration during pre and post-driving tasks [75]. In an emergency, a driver's reaction time is typically seen as sleepiness affected [78]. However, driving studies have shown that a fatigued driver responds to an emergency either typically or not, the latter leading to a crash [79]. In other words, drowsiness disrupts the reaction time of drivers rather than showing a gradual drop [79]. Furthermore, sleeping time is only represented in part of the reactions impacted, typically through momentary lapses [80].

The monotonous roads are considered one of the problems of the driving process. Reacting to response time stimuli can have a remarkable effect, especially when the stimulus is familiar. While it may appear beneficial, this technique masks the underlying drowsiness. If the stimulus is frequent, such as every 30 s, a driver's distraction from the road, even briefly, might be counterproductive [81]. However, extraordinary stimulation cannot have the same impact, making the driver more susceptible to drowsiness (for example, 4−5 min on average). However, the infrequency does not detect tiredness since a critical stimulus may arrive too late to prevent an accident [78]. Their link to accident risk is making reaction time measurements widespread. Many reaction timings may be available, including the number of missed events, improper replies, reaction time, and reaction distance [82]. According to Langner, Steinborn [83], in activities demanding voluntary attention control, reductions in performance because of mental tiredness were more evident. Psychological weariness from extended service times also reduces the time needed to prepare for a quick response. Therefore, it can be inferred that driver reaction time is one of the essential factors that can be considered an indicator(s) to measure driving performance.

Driver vigilance is defined as the ability to maintain concentrated attention over a prolonged driving time [22], while driver attention is defined as any point when the driver engages in a secondary task or looks away from the forward road [84]. The current study found that the attention and vigilance of drivers affect driving performance (β = 0.749, p < 0.000). This finding is in line with previous studies. For example, driver vigilance and attention are critical factors that impact driving performance. Vigilance refers to an individual's ability to sustain attention to detect rare and unpredictable signals for a prolonged time [85]. In the case of driving, vigilance is essential to detect potential collisions and automation failures, especially in automated driving systems [86]. Resource theory provides a possible explanation for the relatively severe vigilance decrement in automated vehicles. Resource theory states that vigilance decrement occurs because individuals have a limited capacity of cognitive information-processing resources that are depleted over time, resulting in insufficient resources to maintain vigilance [85].

Lapses in driving attention contributed significantly (90%) to road accidents [87,88]. Predictions of road accidents are often difficult to identify and are not disclosed to prevent the distribution of blame for numerous incidents. However, 95% of vehicle accidents have been caused by human mistakes [88]. According to Stutts and Hunter [89], driver inattention was estimated at 25–30% of the over 1.2 million traffic accidents recorded yearly in the United States. Higher inattention is a contributing cause of up to 50% of all traffic crashes [88]. Thus, driver attention is associated with road accidents and is a critical risk factor. Memory shortcomings and attention can lead to confusion and impact the driver's performance [90].

Regarding sources of driving distraction (e.g., complicated tools), drivers were proven impaired in noticing and responding primarily to unexpected occurrences [91,92]. Low performance impairs driver concentration and vehicle control decision-making capacity. Tiredness is more prevalent around midnight and in the afternoon than at other times, resulting in drivers losing attention and concentration [93].

In the driving environment, several things can influence alertness. Given the significant role of the careful degradation of the risk of long-haul heavy truck driving, it is essential to identify dependable ways of surveillance and attention for oil and gas drivers. The major vigilance test in this cohort using the psychomotor alert test was one of the vigilance measures utilized in prior studies (PVT) [94]. Concerning the eye-tracking method, blink duration, and frequency increases are related to vigilance decreases [95]. Abe, Nonomura [96] combined a reduction in pupil diameter with increased sleepiness and reduced alertness in the eye closure percentage for a specific time. Vigilance has a role in maintaining the restricted attention needed for the safe, long-distance driving of heavy vehicles [97]. Whether the driver's attentiveness drops due to fatigue, a mental mistake, or a lack of focus, the result is low driving performance [98]. Experience and age impact driver vigilance; less experienced and older drivers may be prone to losing vigilance during driving [99]. Finally, it can be concluded from the above discussion that driver vigilance and attention are considered essential factors to measure driving performance. On the other hand, driving training is essential for drivers to avoid poor driving performance [100].

## 6 Conclusion

The present study investigated driving performance factors among oil and gas tanker drivers. The results showed that drivers' vigilance and reaction time are vital to measuring drivers' performance. Therefore, the study hypothesized that attention to driving performance factors is crucial to avoiding road accidents. Quantitative methods research was conducted through a survey questionnaire to examine these factors. The results of the EFA demonstrated that driving performance could be categorized into two components: reaction time, attention, and vigilance of drivers. Besides, the PLS result showed a significant impact of drivers' reaction time, attention, and vigilance on the driver's performance. This study has several consequences and contributions. The study benefits the oil and gas transportation industry by better comprehending driving performance measures. Second, the study findings establish the groundwork for future studies by confirming that drivers' concern about attention and response time significantly influences driving performance. Third, it gives a strong vision for management oil and gas transportation firms that want to achieve their obligations by improving their driving performance. Finally, the present analysis can assist the parties involved, such as managers, supervisors, and drivers, concentrate on drivers' vigilance and attention throughout oil and gas transportation responsibilities.

### 6.1 Managerial implications

The managerial implications that can be employed by oil and gas transportation management in understanding the impact of driving performance impairment factors on drivers’ awareness of daily driving duties are suggested.

• It provides oil and gas transportation companies with a clear picture that can be leveraged for market competitiveness via awareness of the impact of road accidents on companies' financial losses, assets, and reputation.
• It assists supervisors and managers in evaluating drivers' performance and monitoring their daily activities, allowing for more effective decision-making.
• It provides empirical evidence that may be beneficial in guiding Malaysian authorities and other developing countries to raise awareness of the relevance of driver performance impairment variables and their negative consequences on road safety and national economics.

### 6.2 Theoretical implications

The suggested exploration and model establish a necessity for driver's performance awareness, particularly in developing countries oil and gas transportation industry. This awareness can help overcome obstacles to successful duty delivery in the oil and gas transportation industry. Consequently, the gap in understanding the factors influencing driver performance in the oil and gas transportation sector has been narrowed. Besides, this study has helped in building literature on the subject. Furthermore, this study established the base for future research on driver performance impairment indicators, notably in road safety management. Therefore, the theoretical elements of this study provided an empirical framework for recognizing the driving performance impairment factors that may be used successfully in Malaysia and elsewhere around the world.

### 6.3 Limitations and recommendations for future researchers

Even though this research contributes to academics and practice, such limitations open up new avenues for future research. This study's respondents are comprised of only oil and gas tanker drivers. It is better to get another opinion from the oil and gas companies (supervisors and managers). Therefore, future studies should explore other factors affecting driving performance, such as drivers' distraction factors. In addition, the survey sample comprised oil and gas truck drivers from a single industry. As a result, future research should be expanded to include additional types of heavy trucks.

Furthermore, this study relies on self-report survey data to measure performance and validate driving performance factors. We recommend incorporating objective performance measures like crash records or independently verifiable metrics to improve future studies. Additionally, researchers should consider using experimental methods with advanced technologies, such as the Psychomotor vigilance test and camera detection, for more precise investigations of driving performance factors. Combining self-report data with these objective measures and advanced technology can enhance research methodology and provide a more accurate performance assessment, strengthening the study's conclusions.

## Funding statement

The authors would like to thank Universiti Sains Islam Malaysia (USIM) and Universiti Teknologi PETRONAS (UTP) for funding this research.

## Data availability statement

The data is available for reasonable reasons through contact with the corresponding author.

## CRediT authorship contribution statement

**Al-Baraa Abdulrahman Al-Mekhlafi:** Writing – original draft, Validation, Methodology, Investigation, Formal analysis, Data curation, Conceptualization. **Ahmad Shahrul Nizam Isha:** Writing – review & editing, Visualization, Validation, Supervision, Resources, Project administration, Funding acquisition, Data curation. **Nicholas Chileshe:** Writing – review & editing, Validation, Software, Formal analysis. **Ahmed Farouk Kineber:** Writing – review & editing, Validation, Software, Formal analysis. **Muhammad Ajmal:** Writing – review & editing, Methodology, Data curation. **Abdullah O. Baarimah:** Writing – review & editing, Validation, Software, Methodology. **Al-Hussein M.H. Al-Aidrous:** Writing – review & editing, Validation, Software, Formal analysis.

## Declaration of competing interest

The authors declare that they have no known competing financial interests or personal relationships that could have appeared to influence the work reported in this paper.
